# Impact of Predation by Ciliate *Tetrahymena borealis* on Conjugation in *Aeromonas salmonicida* subsp. *salmonicida*

**DOI:** 10.3390/antibiotics13100960

**Published:** 2024-10-11

**Authors:** Alicia F. Durocher, Valérie E. Paquet, Rébecca E. St-Laurent, Caroline Duchaine, Steve J. Charette

**Affiliations:** 1 Institut de Biologie Intégrative et des Systèmes (IBIS), Université Laval, Québec, QC G1V 0A6, Canadasteve.charette@bcm.ulaval.ca (S.J.C.); 2 Centre de Recherche de l’Institut Universitaire de Cardiologie et de Pneumologie de Québec (IUCPQ), Université Laval, Québec, QC G1V 4G5, Canada; 3 Département de Biochimie, Microbiologie et Bio-Informatique, Université Laval, Québec, QC G1V 0A6, Canada

**Keywords:** conjugation, predation, *Aeromonas salmonicida*, *Tetrahymena borealis*, plasmid, plasmid mobilization, ciliate, furunculosis, antibiotic resistance genes

## Abstract

Background/Objectives: Antibiotic resistance gene (ARG) spread is driven by horizontal gene transfer (HGT). Ciliated protozoa may contribute to this process, as their predation has been shown to facilitate HGT in certain bacteria. Here, this phenomenon was further investigated using *A. salmonicida* subsp. *salmonicida*. This fish pathogen bears an extensive and dynamic plasmidome, suggesting a high potential for HGT. Methods: *A. salmonicida* strains carrying one of three conjugative plasmids bearing ARGs (pSN254b, pRAS1b or pAsa4b) were cocultured with a recipient, either *A. salmonicida*, *E. coli* or *A. hydrophila*. Conjugation rates were assessed in the presence and absence of the ciliate *Tetrahymena borealis*. PCR genotyping confirmed the acquisition of the conjugative plasmids and was used to verify the mobilization of other plasmids. Results: The basal rate of conjugation observed was high. Under the conditions studied, ciliate predation did not appear to influence the conjugation rate, except at higher proportions of ciliates, which typically hampered conjugation. Microscopy revealed that most bacteria were digested in these conditions. PCR screening demonstrated that small mobilizable plasmids from *A. salmonicida* (pAsa1, pAsa2, pAsa3, and pAsal1) were acquired by the recipients along with the conjugative plasmids, with a slight effect of the ciliates in some donor/recipient cell combination. Conclusions: These results highlight how *A. salmonicida* can conjugate efficiently with different species and how complex its relationship with ciliates is.

## 1. Introduction

Ciliated protozoa prey on bacteria, and this predation acts as a selective pressure, driving forward the evolution of bacteria looking to survive grazing [1]. Thus, predation by ciliates has been observed to have a positive impact on certain bacterial strains in specific conditions. Some bacteria can survive digestion by ciliates and gain protection from stresses in the form of bacteria packaging [2], while some present a more virulent phenotype or express genes differentially after going through the phagocytic pathway of ciliates [3]. Furthermore, some species are more resistant to phage infection after being exposed to predation by ciliates [4,5].

Similarly, it has been reported that some bacteria, including *Escherichia coli*, *Salmonella enterica*, and *Aeromonas caviae*, may exhibit an enhanced capacity for conjugation when being preyed upon by ciliates such as *Tetrahymena pyriformis* or *T. thermophila* [6,7,8,9]. Matsushita and collaborators have found that the rate of conjugation between *E. coli* and *A. caviae* increased with the number of ciliates added to the coculture [9]. However, this effect does not appear to extend to other tested protozoa, as no impact on conjugation in *E. coli* was noted in the presence of the amoebae *Acanthamoeba castellanii* or *Dictyostelium discoideum* [7]. Matsuo and collaborators suggested that the influence of *Tetrahymena* on conjugation could be linked to the efficiency with which these ciliates ingest particles [7]. The rapid accumulation of bacteria in phagocytic vesicles seems to favor conjugation, probably by facilitating contact between bacteria. *T. pyriformis* has been found to have an ingestion rate of 80–120% of its cell volume per hour [10]. Additionally, it has been shown that predation by *T. thermophila* supports the maintenance of a conjugative plasmid in *Serratia marcescens* but does not promote the persistence of a non-conjugative version of the same plasmid [11]. Interactions between ciliates and bacteria are complex and vary greatly from species to species [12,13], and it is not yet known whether predation by *Tetrahymena* could affect conjugation in bacterial strains other than those already identified, or if other species of *Tetrahymena* exhibit the same behavior.

*Aeromonas salmonicida* subsp. *salmonicida* is a fish pathogen responsible for furunculosis in salmonids. It is recognized as one of the most important pathogens in fish due to its wide distribution and major economic impact [14]. Furunculosis causes significant economic losses every year, notably in the province of Québec, Canada. In this region, this disease is the most prevalent bacterial infection in fish farms, particularly affecting brook trout, and accounts for 30 to 60% of reported outbreaks annually [15]. The standard treatment for furunculosis is antibiotics; however, this method is becoming increasingly ineffective due to the common occurrence of antibiotic resistance [16,17].

*A. salmonicida* subsp. *salmonicida* is known for its extensive and dynamic plasmidome, which includes many plasmids harboring antibiotic resistance genes (ARGs) [18]. Typically, strains of this subspecies carry multiple plasmids, including what is considered the “standard plasmidome”. This includes large plasmid pAsa5, which bears genes for the type III secretion system (T3SS), an important virulence factor; small plasmid pAsal1, which carries a gene encoding an effector of the T3SS; and small plasmids pAsa1, pAsa2, and pAsa3, which have no known function and are thus considered cryptic [19,20,21]. Specifically, the small plasmids are consistently found in Canadian isolates, while pAsa3 and pAsal1 are more frequently absent in isolates from Europe [22]. It has been suggested that these cryptic plasmids may play a key role in plasticity and adaptation in *A. salmonicida* subsp. *salmonicida* [23]. Moreover, there is evidence of the transfer of ARG-carrying plasmids between *A. salmonicida* subsp. *salmonicida* and other species, including pathogens affecting other animals besides salmonids. This suggests that *A. salmonicida* subsp. *salmonicida* may serve as a potential reservoir of ARGs in the environment [17,24,25].

Previous work by our group suggested that lifestyle significantly influences the fate of *A. salmonicida* when preyed upon by *T. pyriformis*. Mesophilic strains of *A. salmonicida* were found to be more resistant to predation compared to psychrophilic ones [13]. However, since these experiments were conducted at 25 °C to reflect the ideal growth temperature for *T. pyriformis*, it remains unclear to what extent the temperature negatively impacted the resistance capacity of the psychrophilic strains typically found in North America and Europe, which normally grow below 20 °C [26]. Thus, it was considered that a ciliate species more adapted to colder temperatures than *T. pyriformis* should be used in further experiments characterizing the interactions of *A. salmonicida* and ciliates. The species *Tetrahymena borealis*, a protozoan with a lower growth temperature than *T. pyriformis*, was considered a good candidate for this role, as it is widely distributed in North America and Europe, while being part of the *T. pyriformis* complex [27].

This study aimed to evaluate the effect of predation on conjugation in *A. salmonicida* subsp. *salmonicida* and its potential to affect the spread of ARGs. To achieve this, we selected three strains of this bacterium carrying a conjugative resistance plasmid among their plasmidome (pSN254b, pRAS1b or pAsa4b) [24,28,29]. Conjugation assays were performed using these strains as donors and different bacterial species as recipients. *T. borealis* ciliates were added at different ratios to the conjugating pairs to assess their impact on transfer efficiency, considering both the presence of ciliates and the ratio of ciliates to bacteria. The results show a null to negative effect of ciliates on the spread of conjugative plasmids, while revealing the dynamics of ARG-carrying conjugative plasmids as well as small mobilizable plasmids from *A. salmonicida* subsp. *salmonicida*.

## 2. Results

This study aimed to investigate the potential effect of predation by the ciliate *T. borealis* on the conjugation process for various strains of *A. salmonicida* subsp. *salmonicida* through a coculture assay. Three different recipient bacteria were used for the conjugation experiments: *E. coli* DH5α, *A. salmonicida* 19K-308, and *Aeromonas hydrophila* HER1210. Strains used as recipients carried no plasmid before the conjugation assay.

After the conjugation assays, the drop count method on plates with and without tetracycline selection was used to determine the conjugation rate for each condition. Each condition was tested in biological triplicate. Table 1 shows the classification of the conjugation rates observed for each condition, based on the average rate of the three repeats. The individual rates for each repeat of each condition are available in Table S1. The highest rate of conjugation was obtained with *E. coli* DH5α as the recipient for plasmid pRAS1b, at the ciliate ratio C (1:10^6^), with an average conjugation rate of 3.40 × 10^−3^. No conjugation was observed for any of the negative controls, nor for ciliate ratio B (1:10^5^). Cocultures at ratio C showed the most variation between the repeats, while those at ratio D (1:10^7^) showed the least.

**Figure 1:**
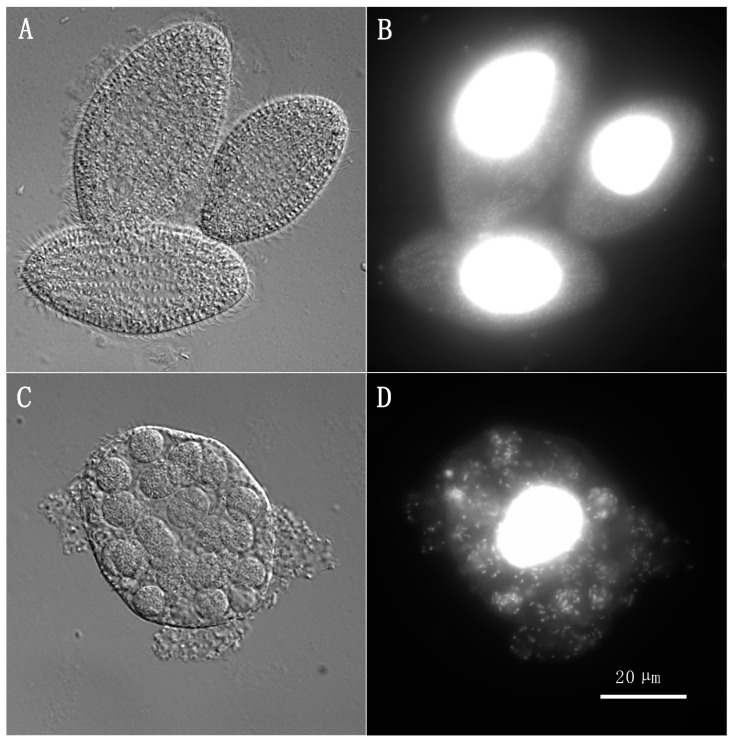
Ciliates/bacteria ratios impact the efficiency of digestion by *T. borealis***.** At ratios A (1:10^4^) and B (1:10^5^) (panels (**A**,**B**), showing a coculture between *E. coli* and *A. salmonicida* subsp. *salmonicida* 2004-05 MF26 at ratio A as an example), digestion was efficient, as evidenced by the absence of bacteria in the medium and in the phagocytic vesicles inside the ciliate cells. At ratio C (1:10^6^) and D (1:10^7^) (panels (**C**,**D**), showing a coculture between *E. coli* and *A. salmonicida* subsp. *salmonicida* 2004-05 MF26 at ratio C as an example), digestion was not as effective, as multiple bacterial cells were still visible in the phagocytic pathway of *T. borealis* cells and outside of those cells. DAPI staining allows the visualization of DNA in epifluorescence (panels (**B**,**D**)). Magnification: 630×.

The observation of samples taken from the conjugation cocultures using differential interference contrast (DIC) and epifluorescence microscopy showed that, at ratios A (1:10^4^) and B (1:10^5^), very few bacteria remained in the medium and the digestive vacuoles of ciliates appeared mostly empty, indicating efficient digestion of the bacteria (Figure 1A,B). At ratios C (1:10^6^) and D (1:10^7^), more bacteria were visible in the samples, both outside and inside the ciliate cells, suggesting that the ciliates were unable to efficiently digest all the bacteria (Figure 1C,D).

**Figure 2:**
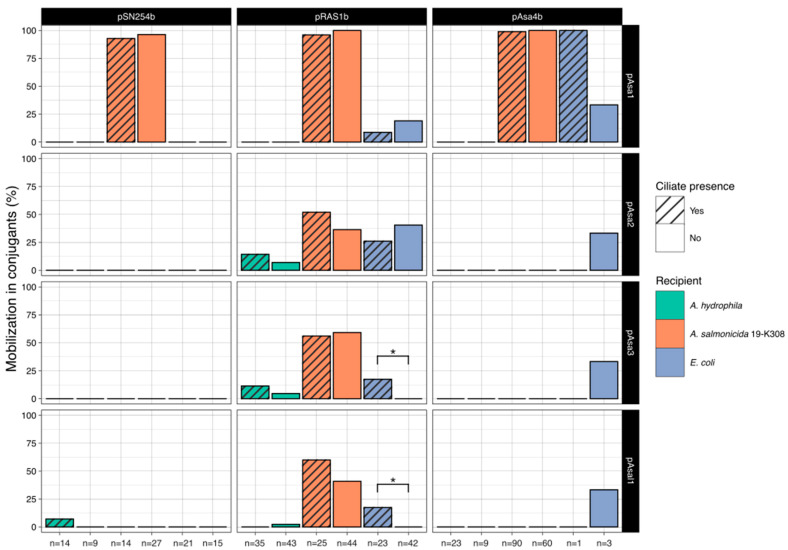
Rates of transfer of small mobilizable cryptic plasmids to conjugants. Shown here are the frequencies of observation of the transfer of the small mobilizable plasmids pAsa1 (first row), pAsa2 (second row), pAsa3 (third row), and pAsal1 (fourth row), as a percentage, as observed through PCR screening of conjugants having also acquired a conjugative resistance plasmid, either pSN254b (first column), pRAS1b (second column) or pAsa4b (third column). Asterisks (*) denote conditions where a significant difference in the proportion of mobilization was observed between the presence and the absence of ciliates (*p* < 0.05). The number of conjugants analyzed for each condition is presented as labels on the *x*-axis and was dependent on the conjugation efficiency in each condition. It should be noted that in the case of *A. hydrophila* having acquired pRAS1b in the presence of ciliates, the number of conjugants analyzed is 30 rather than 35.

Figure 2 shows the frequency of detection through PCR screening of the four small, mobilizable plasmids found initially in donor *A. salmonicida* subsp. *salmonicida* strains and transferred to some of the recipient cells during conjugation. Among the pSN254b transconjugants, out of 100 lysates tested (coming from conditions with or without ciliates), 39% had acquired pAsa1, 1% had acquired pAsal1, and none had acquired either pAsa2 or pAsa3. Out of those 39 conjugants carrying both pSN254b and pAsa1, all were *A. salmonicida* 19-K308. Mobilization of pAsa1 was frequent both in the presence and absence of ciliates.

Among the conjugants having acquired pRAS1b, out of 212 lysates tested, plasmid pAsa1 was found in 37.7% of conjugants, pAsa2 in 28.3% of conjugants, pAsa3 in 23.6% of conjugants, and pAsal1 in 17.9% of conjugants. The small plasmids were found in conjugants of all three strains used as recipients but were more frequently transferred to *A. salmonicida* 19-K308. The plasmids were mobilized both in the presence and absence of *T. borealis*. Mobilization in the presence of pRAS1b exhibited greater variability compared to when pSN254b or pAsa4b enabled the transfer of the small plasmids. Furthermore, pRAS1b showed the most pronounced differences in mobilizable plasmid transfer between ciliate and non-ciliate conditions, although these variations were not substantial.

For the conjugants that acquired plasmid pAsa4b, the only small plasmid consistently detected was pAsa1, found in 81.2% of the 186 conjugants tested, of which all but 2 of the conjugants carrying pAsa1 were *A. salmonicida* 19-K308. Plasmids pAsa2, pAsa3 and pAsal1 were each found in just 1 conjugant out of the 186 screened, representing 0.54% of the conjugants. It should be noted that the mobilization percentages shown for *E. coli* having acquired pAsa4b is based on solely four conjugants. Additionally, all conjugants screened were positive for either pSN254b, pRAS1b or pAsa4b, depending on the assay.

A two-tailed Fisher’s exact test was used to compare the proportion of plasmid mobilization in the presence and in the absence of ciliates for each combination of donor plasmid, recipient bacteria, and mobilizable plasmid, to assess whether ciliate presence could have an impact on plasmid mobilization, be it positive or negative. Only two conditions yielded a *p*-value lower than 0.05: with pAsa3 when transferred to *E. coli* through pRAS1b and with pAsal1, also when transferred to *E. coli* through pRAS1b (*p* = 0.013079 for both). In both cases, the presence of ciliates is associated with a greater mobilization of these small plasmids.

The sequences of the conjugative plasmids used in this study were analyzed using CONJscan to characterize the type IV secretion system (T4SS) carried by each plasmid [30]. The results showed that the T4SS of plasmids pSN254b and pAsa4b belonged to the MPF_F_ class (mating pair formation, type F), while pRAS1b carried a T4SS associated with the MPF_T_ group (mating pair formation, type T). Furthermore, the mobility (MOB) classes of each plasmid were identified through MOBscan [31]. pSN254b and pAsa4b both belonged to the MOB_H_ group, while pRAS1b was classified as MOB_P_.

## 3. Discussion

Interactions between bacteria and ciliates are complex, as once again evidenced by these results. Previous studies had suggested that ciliate predation produced a clear positive effect on conjugation rates in the bacteria studied [6,7,8,9]. However, this does not seem to be the case for *A. salmonicida* subsp. *salmonicida*, at least not when *T. borealis* is the predator. As shown in Table 1, conjugation rates did not increase in the presence of ciliates and were often lower than in their absence, especially at ratios A (1:10^4^) and B (1:10^5^) where conjugation seldom occurred. A previous study investigating the interactions of *A. salmonicida* with *T. pyriformis* demonstrated that the susceptibility and response to grazing of *A. salmonicida* varied greatly from strain to strain [13]. The results suggested that the lifestyle of *A. salmonicida* strains (psychrophilic or mesophilic) could impact the outcome of predation, with psychrophilic strains tending to be digested efficiently by *T. pyriformis*, while mesophilic strains exhibited varying levels of resistance. It is thus understandable that ciliate predation may have a different effect on the bacteria studied here, given the use of a different ciliate and different bacteria, including both psychrophilic and mesophilic strains of *A. salmonicida*.

While the genetic mechanisms explaining the heightened conjugation frequency due to predation previously reported have not been identified, Matsushita and collaborators have shown that contact between the bacteria and the ciliate was necessary for this effect to occur. Separating the bacteria and the ciliates by using a transwell insert led to a decrease in the rate of conjugation, and they showed through fluorescence microscopy that the donor and recipient strains co-localized in the digestive vacuoles of *Tetrahymena* [9]. This forced proximity between donor and recipient is thought to favor cell-to-cell contacts and thus conjugation. It has also been highlighted that predation by protozoa may indirectly favor conjugation by keeping bacterial populations from reaching their stationary phase, where conjugation is less likely [11].

It has been previously suggested that temperature may have an impact on the efficiency with which ciliates facilitate conjugation. A stronger effect on conjugation between strains of *E. coli* was observed with *T. pyriformis* and *T. thermophila* when cocultures were incubated at 30 or 37 °C [7]. However, as our experiment focused on ciliates as a tool for transfer in *A. salmonicida* subsp. *salmonicida*, we chose to conduct the conjugation assay at a temperature more reflective of growth conditions for this bacterium. Thus, we used its ideal growth temperature of 18 °C for coculture incubation. The isolate of *T. borealis* selected was originally found in a pond in Long Lake, Michigan, which we believed would be more representative of ciliates found on North American fish farms based on climate. This temperature should not hinder growth in recipient cells, at least for mesophilic *Aeromonas* considering that their growth capacity at 18 °C is even better than that of psychrophilic *A. salmonicida* subsp. *salmonicida* [32,33].

The highest rate of conjugation observed in this study was when *E. coli* DH5α served as the recipient for plasmid pRAS1b, at ratio C (1:10^6^). This specific condition was the only one that differed noticeably from the basal conjugation rate in the absence of ciliates, being tenfold higher. As *E. coli* DH5*α* is a lab-engineered strain [34], these results may not be as reflective of the conditions that would be observed in Québec fish farms as the results obtained with strain *A. salmonicida* 19-K308, which was isolated from this precise environment. However, since *E. coli* DH5*α* had already been shown to be capable of receiving pSN254b from *A. salmonicida* subsp. *salmonicida* through conjugation [35], it was included in our study as a tool to validate the conjugation assay, by having at least one condition where conjugation was known to be possible. Interestingly, the rate of conjugation observed by McIntosh et al. was higher than the one seen in this experiment. This discrepancy could be explained by the different conjugation protocols used.

As shown through analysis with CONJscan, both pSN254b and pAsa4b harbored a T4SS of class MPF_F_. MPF_F_ systems of conjugation are described as complex and allow mating in liquid conditions. In contrast, pRAS1b carries a T4SS of class MPF_T_, which is seen as a precursor of MPF_F_ systems, simpler and adapted only for surface mating [36]. We considered that using a plasmid carrying this type of MPF system could help us better understand the mechanisms behind the possible effect of ciliates on conjugation, as *Tetrahymena* could only have a direct effect on conjugation in a liquid medium, as suggested by previous evidence indicating that this effect was due to forced cell-to-cell contacts in the ciliates’ digestive vacuoles [9]. However, the highest rate of conjugation seen in our study was obtained with this plasmid, pRAS1b, at ratio C (1:10^6^) with *E. coli* as the recipient, which could possibly indicate that ciliates might still influence conjugation, despite the conjugation mostly occurring during incubation on TSA. It has previously been reported that bacteria surviving ciliate predation could present changed phenotypes [4,37], so it is not out of the question that a similar mechanism could be at play in this case, but further investigation would be necessary.

The rates of conjugation observed at ratios A and B (10^4^ or 10^5^ bacteria for every ciliate, respectively) were typically null, except for some iterations of the triplicates for certain conditions at ratio A, such as when pSN254b was conjugated. These results may be explained by microscopy observations (Figure 1A,B), which showed very little survival of bacteria in these conditions and efficient digestion of the bacteria consumed after 24 h, as previously observed with another ciliate in the past [13]. At these ratios, rates of conjugation are then often null since the possibility of conjugation diminishes as the number of surviving donor and recipient bacteria decreases. However, when conjugation does occur, the low number of surviving recipients results in a very high rate of conjugation. Ultimately, in our experiment, it seems that ciliates do not favor conjugation for the bacterial strains studied and can have, in fact, a negative effect on it, as seen with ratios A (1:10^4^) and B (1:10^5^). Based on these results, bacterivorous ciliates could then be considered as a potential method of control for the transfer of ARGs through conjugation in fish farms dealing with resistant *A. salmonicida* subsp. *salmonicida*. Previous results had already shown a low rate of survival to ciliate predation for psychrophilic strains of *A. salmonicida* subsp. *salmonicida*, which represent most problematic strains for Québec fish farms [13]. However, it is important to note that some ciliate species are major parasites of fish, and that some *Tetrahymena* species have been described as histophagous and opportunistically pathogenic [38,39,40]. Strains of *Tetrahymena thermophila* have been shown to consume a monolayer of cells from salmonid fish [41]. Therefore, further research is needed to assess the potential use of ciliates as a solution against ARG transfer in fish farms.

The basal rates of conjugation observed during the conjugation assay are high when compared to the values found in other similar experiments [7,9]. This evidence points to *A. salmonicida* subsp. *salmonicida* having a strong potential for conjugation in its environment, which is already favorable to horizontal gene transfer (HGT) [42]. This high rate of conjugation also supports the growing idea that this bacterium could act as a reservoir of plasmids and ARGs [16].

An interesting result observed in this study was the difference in the mobilization of the small cryptic plasmids depending on the strain that acted as a donor in the conjugation assay, and thus depending on the conjugative plasmid that enabled mobilization (Figure 2). For the conjugation assays with strain 2004-05 MF26, where pSN254b was the conjugative plasmid that could potentially allow the transfer of the small mobilizable plasmids, only pAsa1 was transferred, and only to recipient strain *A. salmonicida* 19-K308. However, when strain 2004-072 acted as the donor in the conjugation assay, plasmid pRAS1b allowed the mobilization of all four small plasmids to varying degrees. Although the plasmids seemed more readily transferred to strain 19-K308, some conjugants from all three recipient strains acquired pAsa2 and pAsa3. In the case of pAsa1, no *A. hydrophila* lysate analyzed received it. With strain SHY13-2627 carrying pAsa4b, similar results to those seen with pSN254b were obtained. pAsa1 was the only small plasmid consistently mobilized, although one *E. coli* conjugant acquired all small plasmids along with pAsa4b. Since very little conjugation was observed with *E. coli* for pAsa4b, we were not able to collect more conjugants for analysis.

The difference in mobilization could be explained by the different classes of mobility (MOB) and mating pair formation (MPF) genes found on all these plasmids. pSN254b carries the relaxase gene *mobH*, which belongs to the MOB_H_ class of relaxases [24,36]. This family was noted to be one of the least commonly found in the plasmids studied by Smillie et al. and to be mostly associated with large plasmids, which is consistent with pSN254b being a 152 216 bp plasmid. pAsa4b, the other large plasmid used in this study, was also found to belong to the MOB_H_ group based on analysis of its relaxase through MOBscan [31]. As for pRAS1b, pAsa1, pAsa2, pAsa3, and pAsal1, they are all classified into the MOB_P_ family of relaxases [31,36]. pAsa1, pAsa2, pAsa3, and pAsal1 carry the *mobA* relaxase gene, while pRAS1b seems to carry *virD2* [20,28,30]. As the relaxases of pRAS1b and the small plasmids belong to the same class, the MPF complex of pRAS1b may be more apt to transfer these plasmids than the MPF complexes of pSN254b or pAsa4b. It should also be noted that the T4SS of pRAS1b belongs to the MPF_T_ group, which is associated with plasmids showing a broad host range [36]. It may be possible that the characteristics of the plasmid that permit its wide range of hosts could also allow the mobilization of more plasmids that lack an MPF module. In the same way, MPF_F_ plasmids are described as more complex, which could possibly allow a better control of mobilization, explaining the more restricted mobilization of the small plasmids seen here [36].

It was also noted that pAsa1 is the only plasmid able to be reliably mobilized by pSN254b, pRAS1b, and pAsa4b, or at least to be replicated in the receptor cells following mobilization. When first characterized, pAsa1, pAsa2, and pAsa3 had all been described as having an *oriT* site resembling that of other pColE1-adjacent plasmids [20]. Our data suggest that the *oriT* site of pAsa1 is sufficiently different from the others to be the only one recognized by the T4SS and the type IV coupling protein (T4CP) of pAsa4b and pSN254b. The T4CP is identified as a key agent in the specificity of the conjugative pore [43].

Altogether, pRAS1b allowed mobilization in the most diverse conditions. Some effect on mobilization from the presence of ciliates was observed with pRAS1b, unlike when another conjugative plasmid permitted mobilization. The only two conditions that yielded a significant *p*-value through a two-tailed Fisher’s exact test when comparing ciliate presence and absence both had pRAS1b as the conjugative plasmid and *E. coli* as the recipient, for pAsa3 and pAsal1. For these two conditions, the null hypothesis could be rejected, and ciliate presence could be said to influence mobilization of the small plasmid. However, as stated previously, *E. coli* DH5*α* is not the most environmentally relevant recipient strain, so the reach of this effect in natural settings should be studied further.

The high rate of transfer of the small cryptic plasmids observed here may further explain their wide distribution in *A. salmoncida* subsp. *salmonicida* isolates despite their carrying no known function. In 2008, a study of the plasmidome of bacteria showing reduced susceptibility to antibiotics in a wastewater treatment plant sample identified these small plasmids as being abundant in the sample. While the plasmids themselves carry no ARGs, they were still the most frequently found plasmids in the study [44]. Cryptic plasmids have been identified as a possible “tool” for adaptability and evolution in *A. salmonicida* subsp. *salmonicida* [23].

## 4. Materials and Methods

### 4.1. Ciliates and Bacteria

The *Tetrahymena* strain used was *T. borealis* 20754-1 (*Tetrahymena* Stock Center, Cornell University), isolated from Long Lake, Michigan. *T. borealis* was grown axenically in modified NEFF medium (0.25% proteose peptone, 0.25% yeast extract, 0.5% glucose, and 33.3 µM of FeCl_3_ [45]) at 18 °C. The medium was supplemented with 250 µg/mL of streptomycin, 250 µg/mL of penicillin G, and 1.25 µg/mL of amphotericin B. The cells were subcultured upon reaching confluence.

The bacterial strains used in this study are listed in Table 2. For bacteria, stock cultures were stored at −70 °C in lysogeny broth (LB) medium (Wisent, St-Bruno, QC, Canada) supplemented with 15% glycerol. Before each experiment, the stock cultures were thawed and inoculated on Tryptic Soy Agar (TSA) (Wisent, St-Bruno, Canada) plates, and incubated for 72 h at 18 °C. Donor strains were selected for their possession of a conjugative plasmid with ARGs and their psychrophilic lifestyle. All recipient strains used were plasmid-free and exhibited a mesophilic lifestyle which was used as a counter selection of psychrophilic donor cells by incubating bacteria at 37 °C after conjugation [29,35] since they do not present usable antibiotic resistance for counter selection. These mesophilic strains also grow well at 18 °C [33].

### 4.2. Cocultures and Conjugation Assay

Before each conjugation assay, the bacterial strains were inoculated in 10 mL of Tryptic Soy Broth (TSB) (Wisent, St-Bruno, Canada) and incubated overnight at 18 °C with shaking at 200 revolutions per minute (rpm). These cultures were then subcultured and incubated at 18 °C with shaking at 200 rpm for 4 h. The OD_600_ of the cultures was measured and the volume of each culture was adjusted to obtain a concentration of 10^9^ cells/mL in Page’s Amoeba Saline (PAS) [46]. In parallel, a confluent subculture of axenic *T. borealis* (about 10^5^ cells/mL) was enumerated using a hemacytometer. The cells were progressively washed by centrifugation to replace the NEFF medium with PAS. The ciliates were resuspended in an appropriate volume of PAS to obtain a concentration of 10^6^ cells/mL. Serial dilutions of this ciliate suspension were prepared using PAS to obtain concentrations of 10^5^, 10^4^, and 10^3^ cells/mL.

Cocultures for the conjugation assay were prepared in a 24-well plate as follows, and as detailed in Table 3. Each coculture contained 5 × 10^8^ CFU of one of the donor bacteria, 5 × 10^8^ CFU of one of the recipient bacteria, and 100 µL of one of the four dilutions of ciliates: 10^6^ cells/mL for ratio A, 10^5^ cells/mL for ratio B, 10^4^ cells/mL for ratio C, and 10^3^ cells/mL for ratio D. For each donor–recipient combination, a control without ciliates was prepared to assess the basal conjugation rate (basal rate control). A negative control, containing no ciliates and no donor bacteria, was prepared for each recipient strain to verify the absence of natural resistance to tetracycline. The cocultures were then incubated at 18 °C for 24 h.

After incubation, samples of the cocultures were taken for DAPI staining and observation (see below). Triton X-100 was then added to each coculture to a final concentration of 0.1%, to lyse the ciliates and free any intracellular bacteria. After 1 min, ciliate lysis was confirmed through phase contrast microscopy. Once lysis was assessed, the cocultures were harvested and washed twice in PAS buffer to remove all traces of Triton, ensuring it did not affect bacterial viability. Serial dilutions of the cocultures, up to 10^−6^, were prepared in PAS buffer. A drop count was performed by plating 10 µL drops of all dilutions from 10^0^ to 10^−6^ on both TSA and TSA +5 µg/mL tetracycline plates, since all conjugative plasmids studied carried a tetracycline resistance gene (either *tetA* or *tetA* (E)). The tetracycline concentration used was previously established for conjugation of these plasmids. The plates were then incubated for 20 h at 37 °C, after which the colonies on each type of medium were counted. The rate of conjugation was determined by dividing the titer of conjugants, as observed on the tetracycline plates, by the titer of total survivors for the recipient strain, as obtained on the TSA plates. All coculture assays were conducted in a biological triplicate.

### 4.3. PCR Genotyping

After the conjugation experiments, transconjugants from each condition were selected for confirmation by PCR. Isolated colonies of each transconjugant were inoculated with a sterile toothpick onto fresh selective medium then incubated for 24 h at 37 °C. Lysates of these transconjugants were prepared by adding a portion of the transconjugant colony to 20 µL of SWL lysis buffer (50 µM of KCl, 10 µM of Tris Base, 53.4 µM of MgCl_2_, 0.45% of Tween 20, and 0.45% of NP40, in 50 mL of water), followed by incubating for 15 min at 95 °C. Lysates were then stored at −20 °C until the PCR experiments, which were conducted as previously described [22]. The primers used are detailed in Table 4. The PCR screening was used to confirm the presence of pSN254b, pRAS1b or pAsa4b, respectively, in suspected conjugants, and to determine whether other mobilizable plasmids (pAsa1, pAsa2, pAsa3, and pAsal1) present in the donor strains were also transferred along with the conjugative plasmid. A two-tailed Fisher’s exact test was used to compare the proportions of mobilizable plasmid acquisition in the lysates screened between ciliate and no ciliate conditions.

### 4.4. DAPI Staining and Microscopy

Coculture samples were taken after the 24 h incubation, prior to lysis with Triton X-100, and mixed with 8% paraformaldehyde (PFA, final concentration: 4%) for 20 min for fixation. PFA was replaced by centrifuging the samples at 10,000 rpm (9600× *g*) for 2 min, then washing the pellets with 40 mM of NH_4_Cl in 1X phosphate-buffered saline (PBS), followed by a second wash with 1X PBS. The PBS was then replaced by 2.5 µg/mL of DAPI in 1X PBS. The samples were incubated in the dark for 30 min, followed by 2 more washes in 1X PBS. After centrifugation, the pellets were resuspended in 10–50 µL of 1X PBS. Ten microliters of the stained sample were mixed with 10 µL of Prolong Gold (Invitrogen, Whitby, ON, Canada) on a microscope slide, before sealing a coverslip on top. The prepared slides were observed with a Zeiss Axio Observer Z1 microscope equipped with an Axiocam MRm camera (Carl Zeiss, North York, ON, Canada) using differential interference contrast (DIC) and epifluorescence.

### 4.5. Sequence Analysis of Conjugative Plasmids

The sequences of the conjugative plasmids used in these experiments, which were already available on GenBank (accession numbers: NZ_KJ909290.1, NZ_MW033206.1, NZ_KT033469.1), were analyzed using MOBscan and CONJscan to identify or classify the genes involved in conjugation for each of the plasmids [30,31].

## 5. Conclusions

The results of this study offer new insight into the role of *A. salmonicida* subsp. *salmonicida* as a reservoir of ARGs. They also enhance our understanding of the mobility of this bacterium′s plasmidome, which has been known to be dynamic due to numerous evidence of transfer with other species. Based on our results, *T. borealis* does not effectively promote conjugation in this bacterium and may even hinder the process when predation activity is high. Ciliates could be considered as a method of control to mitigate the spread of problematic plasmids on fish farms. However, adding ciliates to fishponds may not be a viable solution, as the ciliate selected may not be adapted to the environment or may disrupt established microbial communities. Instead, fostering diverse communities in fish farms, including ciliates native to this environment, may be a more sustainable approach.

Additionally, our study is the first to demonstrate that the small plasmids of *A. salmonicida* subsp. *salmonicida* can be strongly mobilized during conjugation, particularly in the presence of the pRAS1b plasmid. The role of the ciliate in this context appears to be minor and is significant only under very specific and unpredictable conditions.

## Figures and Tables

**Table 1:** Conjugation frequency of pSN254b, pRAS1b, and pAsa4b with different recipients in the presence and absence of ciliates. Ratios A to D refer to cocultures which included *T. borealis*, while the basal rate control contained only the recipient and donor strains, and the negative control included only the recipient strain. Due to considerable variation between repeats (Table S1), the color of each cell indicates the consistency of the results. The detailed meaning of the code and the colors used is available in the table footnote.

Plasmid	Recipient	Ratio A 1:10^4^	Ratio B 1:10^5^	Ratio C 1:10^6^	Ratio D 1:10^7^	Basal Rate Control	Negative Control
pSN254b	*A. hydrophila*	−	−	++	+++	++	−
	*A. s.* 19-K308	+++	−	++	++	++	−
	*E. coli*	+++	−	+++	+++	+++	−
pRAS1b	*A. hydrophila*	−	−	++	++	++	−
	*A. s.* 19-K308	−	−	+++	+++	+++	−
	*E. coli*	−	−	+++	+++	+++	−
pAsa4b	*A. hydrophila*	−	−	+	+	+	−
	*A. s.* 19-K308	−	−	++	++	++	−
	*E. coli*	−	−	+	−	−	−

Footnote: The average frequency of conjugation for each condition is indicated by a symbol grade: +++ means a conjugation rate above 1 × 10^−4^, ++ means a conjugation rate between 9.99 × 10^−5^ and 1 × 10^−6^, + means a conjugation rate under 1 × 10^−6^, and − means that no conjugation was observed for this condition. The color of each cell in the table indicates the variation in the outcome of the assays (n = 3) for each condition. Dark gray cells signify that none of the three assays yielded conjugants; blue cells indicate that one of the three assays yielded conjugants; yellow cells indicate that two of the three assays yielded conjugants; and white cells denote conditions for which all assays produced conjugants.

**Table 2:** Characteristics of the bacterial strains used in the conjugation assay.

Strain ^a^	Role in Conjugation Assay	Lifestyle	Origin	ARGs Carried by Conjugative Plasmid
*A. salmonicida* subsp. *salmonicida* 2004-05 MF26	Donor (plasmid pSN254b)	Psychrophilic	New Brunswick, Canada [24]	*tetA*, *floR*, *sul1*, *sul2*, *blaCMY*, *aadA*, *strA*, *strB*
*A. salmonicida* subsp. *salmonicida* 2004-072	Donor (plasmid pRAS1b)	Psychrophilic	British Columbia, Canada [28]	*tetA*, *sul1*
*A. salmonicida* subsp. *salmonicida* SHY13-2627	Donor (plasmid pAsa4b)	Psychrophilic	Québec, Canada [29]	*tetA*(E), *sul1*
*A. salmonicida* 19-K308	Recipient	Mesophilic	Québec, Canada [33]	N/A
*A. hydrophila* HER1210 (ATCC 7966)	Recipient	Mesophilic	ATCC/Félix d’Hérelle reference center	N/A
*E. coli* DH5α	Recipient	Mesophilic	[34]	N/A

**^a^** The donor strain carries other plasmids in addition to the conjugative one. In all cases, they bear pAsa1, pAsa2, pAsa3, pAsal1, and pAsa5.

**Table 3:** Composition of the cocultures used in the conjugation assay. The ratios indicate the number of bacteria (donor and recipient combined) for each ciliate cell.

	Ratio A 1:10^4^	Ratio B 1:10^5^	Ratio C 1:10^6^	Ratio D 1:10^7^	Basal Rate Control	Negative Control
Donor bacteria	*A. salmonicida* subsp. *salmonicida* (2004-05 MF26, 2004-072 or SHY13-2627) (5 × 10^8^ CFU)					N/A
Recipient bacteria	*A. salmonicida* 19-K308, *A. hydrophila* or *E. coli* (5 × 10^8^ CFU)					
*T. borealis*	10^5^ cells	10^4^ cells	10^3^ cells	10^2^ cells	N/A	

**Table 4:** Primers used for PCR genotyping.

Target	Forward Primer Sequence	Reverse Primer Sequence	Reference
pSN254b (*blaCMY* gene)	GACAGCCTCTTTCTCCACATTTGC	CTGGTCATTGCCTCTTCGTAACTC	This study
pRAS1b (backbone)	TGATATGGACAGCCACAAATG	CTTCACCGATCACGTCTTTG	[28]
pAsa4b (backbone)	ACTTATAAGACCATCCTGACGGC	TGAGATATCTTTCCAGTCCACACC	[29]
pAsa1 (toxin- antitoxin region)	GGACGATTAACCTTCGCATC	GTATCGCCCAACTTCTTCCA	[20]
pAsa2 (hypothetical protein gene)	AAAAGAGCGTGCAACCCTAA	GCGATGCTACTTCATTCACC	[20]
pAsa3 (toxin- antitoxin region)	TCATGGAGAATGTTCGCAAG	GCCCAATTATCACAGCAACA	[20]
pAsal1 (*aopP* gene)	TAACATGGGTGAGTCAGGA	TGCATGTTTGTAAAAAGTAGGTG	[20]

## Data Availability

The original contributions presented in this study are included in the article/Supplementary Materials, further inquiries can be directed to the corresponding author/s.

## Supplementary Materials

The following supporting information can be downloaded at https://www.mdpi.com/article/10.3390/antibiotics13100960/s1, Table S1: Rates of conjugation observed for pSN254b, pRAS1b, and pAsa4b with different recipients in the presence and absence of ciliates.
